# Comparing exercise interventions to increase persistence with physical exercise and sporting activity among people with hypertension or high normal blood pressure: study protocol for a randomised controlled trial

**DOI:** 10.1186/1745-6215-15-336

**Published:** 2014-08-28

**Authors:** Chris Fife-Schaw, Simon de Lusignan, Joe Wainwright, Hannah Sprake, Suzannah Laver, Victoria Heald, Julian Orton, Matt Prescott, Helen Carr, Mark O’Neill

**Affiliations:** School of Psychology, University of Surrey, Guildford, GU2 7XH UK; Department of Health Care Management and Policy, University of Surrey, Guildford, GU2 7XH UK; Surrey Clinical Research Centre, University of Surrey, Guildford, GU2 7XP UK; Active Surrey, Quadrant Court, 35 Guildford Rd, Woking, GU22 7QQ UK; Public Health, Surrey County Council, Room G55 County Hall, Penrhyn Road, Kingston upon Thames, KT1 2DN UK; Spring Street Surgery, Bourne Hall Health Centre, Ewell, KT17 1TG UK; Isostasy, 3 Riverside, London Street, Whitchurch, Hants, RG28 7LW UK

**Keywords:** Sports, Exercise referral, Hypertension, Web-based self-help tool, Health behaviour change

## Abstract

**Background:**

Increasing physical activity is known to have health benefits for people with hypertension and related conditions. Current general practitioner referrals for gym-based exercise increase physical activity but meta-analyses show that while these are effective the absolute health risk reduction is small due to patients failing to maintain activity levels over time. This study assesses the effectiveness of two sports-oriented interventions that are intended to bridge the intention-behaviour gap and thus increase the likelihood of sustained increases in physical activity.

**Methods/design:**

Four-arm randomised controlled trial. The study tests two types of intervention that are intended to increase physical activity among currently inactive 18- to 74-year-old people with hypertension or high-normal blood pressure. This study will assess the effectiveness of a 12-week sports-oriented exercise programme, the efficacy of a web-delivered self-help tool to promote and support sports participation and healthy behaviour change and the effect of these interventions in combination. The control arm will be a standard care general practitioner referral for gym-based exercise. Participants will be allocated using block randomisation. The first author and primary analyst is blinded to participant allocation. The primary outcome measures will be time spent in physical activity assessed in metabolic equivalent minutes per week using the International Physical Activity Questionnaire 1 year after commencement of the intervention. Secondary outcomes include increased involvement in sporting activity and biomedical health outcomes including change in body mass index, and waist and hip measurement and reductions in blood pressure.

**Discussion:**

If proven to be superior to general practitioner referrals for gym-based exercise, these sports-oriented interventions would constitute low-cost alternatives. The next stage would be a full economic evaluation of the interventions.

**Trial Registration:**

Current Controlled Trials ISRCTN71952900 (7 June 2013).

## Background

The health benefits of sport and physical activity (PA) have been studied extensively. The results demonstrate that participation in PA, which includes sporting activity, is associated with a reduced risk of over 20 health conditions, including cardiovascular disease and some cancers [1, 2]. Targeting those with the greatest levels of inactivity will subsequently have an effect on all-cause mortality [3] and produce the highest decrease in the incidence of chronic disease. In the most recent Cochrane review of interventions intended to increase physical activity [4], the authors conclude that: "Nevertheless interventions which provide people with professional guidance about starting an exercise programme and then provide ongoing support may be more effective in encouraging the uptake of physical activity. There is no evidence that such interventions will reduce physical activity or cause other harm. There is only very limited evidence of the long-term effectiveness of interventions” (page 12).

Individuals report numerous barriers that impede their ability to be physically active [5]. Lack of time and access to facilities are among the most common barriers that have been identified. “Exercise on prescription” interventions that involve a health professional’s written advice to a patient to be more physically active have been used with variable success [6, 7]. A meta-analysis of the efficacy of gym-based exercise referrals for inactive people with medical conditions [8] suggested that while these were effective the absolute health risk reduction was small due to patients failing to maintain activity levels over time. The authors show that, across the studies, 17 sedentary people would have to be referred for one person to become moderately active. They cite a range of barriers such as a lack of self-efficacy, poor body image, poor time management, lack of social support, intimidating environments, inadequate supervision and inconvenient opening hours as limiting the efficacy of these interventions.

Given the lack of time and potential access limitations, it is important to find ways, other than face-to-face programmes, to provide individuals with information, skills, and knowledge to facilitate behaviour change. This study looks at the efficacy of two interventions intended to overcome some of the barriers identified above, both with a focus on making activity fun by linking it with entry-level sporting activity. The first is a web-based behaviour change support tool that allows users to easily find out about outlets for organised activity, form and store plans to carry out the activities and to form a log of what they have achieved. The tool provides educational material and easy access to existing health maintenance sites. The second is a 12-week sports-oriented exercise programme intended as a direct substitute for existing gym-based general practitioner (GP) exercise referral. As with the latter this will be supervised by a qualified exercise professional but participants are given a choice of entry-level sports to pursue. Sports offered will include squash, badminton (‘Badmintone’), netball (‘Netfit’), tennis, swimming, walking football, bowls, athletics (‘AthleFIT’), ‘Cardio Tennis’ and a new ‘Healthy Cycle Ride’. These activities are appropriate for participants with controlled hypertension and, in line with the Surrey Exercise and Weight Management Referral Scheme Protocol, the exercise referral professional will take into consideration any other medical conditions of participants to ensure the sessions are designed and delivered in a safe and effective manner.

Using ideas from social cognition models, both interventions introduce elements of personal choice into activity planning. Perceptions of control are known to facilitate maintained behavioural change and people are known to be more likely to continue to maintain healthy lifestyles if they have freely chosen activities than if they have been instructed to carry them out [9]. This is a feature incorporated in both interventions. The current gym-based referral lacks this element of choice, and by introducing the fun element of sports and games the intention is that participants will become more engaged in their activity. By giving people a choice of sports/games they can choose whether to get involved in games involving others, thus adding a social element to the activity, or they can choose sport activities that can be performed on a more individual basis (for example, cycling, swimming) if preferred.

Similarly, social cognition models highlight the importance of the formation of implementation intentions and these are a central feature of the web-based intervention. Research in this field [10] shows that good intentions to change behaviour patterns often fail as a result of people failing to make the necessary intermediate plans to achieve their goals. For example, someone may intend to go running after work but fail to do so as they have left their running shoes at home.

### The interventions

#### Interactive web tool

The internet can serve as a useful tool in providing health information to large numbers of individuals [11] and there is evidence that among working-age adults the internet is increasingly becoming a preferred method of obtaining health information [12]. There is a plethora of data supporting the use of web-based interventions for behavioural change [13, 14] and to increase PA [15–18]. Davies and colleagues’ recent meta-analysis [16] of internet-delivered PA interventions stated that the inclusion of educational components significantly increased intervention effectiveness. Results of this meta-analysis support the delivery of internet-delivered interventions in producing positive changes in PA, especially in those who are initially inactive, though the number of studies of the initially inactive remains small. None of the studies reported explicitly targeted inactive patients with hypertension and those that targeted overweight patients tended to have relatively small samples (all less than 221).

The tool is intended to mimic a section of a GP practice website that promotes health and wellbeing. Access during the study is encouraged on a regular basis. There is evidence that changing one health risk behaviour can facilitate improvement in a second health behaviour (‘success breeds success’). Additionally, increased confidence (self-efficacy) in changing one behaviour has been shown to be associated with increased confidence in changing a second behaviour [19]. The web intervention directs patients in the website arms to interact with a web-delivered PA intervention which is based on the principles of *Let’s Get Moving*, a public health pathway designed to increase levels of PA through individual behaviour change. There is also the opportunity to browse the host health and wellbeing website and secondarily self-refer to other web-delivered health behaviour interventions (smoking, alcohol and diet). The rationale for this type of interaction is that by allowing self-selection and sequencing of interventions over time, we will achieve positive reinforcement of the PA intervention through capture of the benefit of change in other areas of health risk behaviour, whilst avoiding the danger of ‘overloading’ patients with too much information and too many competing priorities as in a conventional, simultaneous multiple health behaviour intervention.

Increased PA will also be encouraged by means of the general information and support provided throughout the site (including a focus on incorporating PA into daily routines).

Self-selection and self-sequencing of behaviour change goals and pathways will help align the intervention with the patient’s motivations, supports principles of behaviour change such as autonomy, choice and ‘interactivity’ at a primary level, and is consistent with the evidence base on effectiveness of this type of intervention [16].

The tool is interactive and allows patients to make plans and form implementation intentions (the detail when, where, with whom, and so forth; decisions required to enact a more general plan) and draw on information on sports availability and other health-related self-care sites. It draws on some existing UK-based web resources (for example, *Let’s Get Moving*, SPOGO online sports and physical activities database, Live Well, Active Surrey) and the main emphasis is on integrating resources into an attractive interactive site that links health care professionals to signposting of sporting opportunities.

The patient is given control over the means and frequency of prompts and reminders (either via email or text message, and so forth) and be able to create, store and review action plans. The tool uses database information to personalise reminders and identify local activity opportunities. For example ‘John’ might declare an intention to cycle more and the ‘reminder’ function will alert John to any upcoming organised cycle rides in his locality. Patients will have the facility to download plans and activity logs that they can show their GP or share with friends if they wish. To better understand people’s behaviour when using the web tool we will monitor click through rates and other indices of engagement with the tool.

#### Sports exercise referral

The evidence of the effectiveness of interventions aimed at promoting sporting activity is of mixed quality. A recent Cochrane review reported that no controlled trials existed to assess the impact of sports interventions on sports participation [20]; however, Cavill and colleagues in a review of studies with less restrictive design criteria (for example, controlled before and after studies) are able to point to a number of studies that show positive effects of structured sports training sessions on physical and sporting activity levels [1]. They report nine studies in which sporting interventions have led to greater participation primarily through making activity enjoyable.

Underpinning theorising about the motivations to be active are notions of utility and hedonic calculus relating back to the 18th century ideas of Jeremy Bentham [9, 21]. The basic assumption is that if exercise is pleasurable people will be more likely to repeat the experience and conversely if past experiences of exercise are unpleasant then the motivation to repeat the experience will be diminished. Although exercise is often promoted as having positive effects on mood it is far from clear that all people experience this positive outcome, particularly when pressed to exercise on gym equipment as is the case in GP exercise referrals. Indeed in the last decade or so a number of studies have shown that many people experience negative affective states, especially if they exercise at intensities approaching their ventilatory threshold [22–25]. It has also been shown that even at intensities below the ventilatory threshold there is considerable variability in the degree of positive affect experienced during exercise which, in turn, has been implicated as a reason why activity levels remain low for many people.

The challenge is to associate exercise with other affective responses such as those resulting from success or socialising – hence an interest in linking activity to sport rather than repetitive activity on gym equipment. Several studies [26, 27] have shown that affective responses within an exercise session are predictive of subsequent exercise behaviour at 6 and 12 months in sedentary adults, suggesting that the nature of the experience of exercise is important in determining future exercise maintenance. Similarly it has been shown that allowing people to exercise at self-determined intensities increases affective responses during exercise and the likelihood of long-term maintenance of exercise activity [9]. This work shows that perceptions of choice and autonomy appear to be important factors in maintaining behaviour change.

In the proposed sports exercise programme, patients will be given access to a timetable of supervised sports activities that will be available to them at a variety of dates and times in the week; for example, sessions will be available during peak and off-peak hours as well as weekends and weekdays. Patients will be able to book sessions in advance and also turn up to sessions without booking. A variety of sports activities will be included as shown in the list provided previously. Each session will be 30 minutes to 1 hour long and supervised/delivered by a qualified instructor, and will follow national guidelines for exercise referral [28]. The sports sessions will be based around an informal and relaxed version of the sport which has been developed over the last few years by the relevant National Governing Body. For example, the tennis sessions will be based around the popular Cardio Tennis sessions which encourage participants to perform well-known tennis based actions (for example, forehand shots) at a reasonable intensity to music without the use of balls. This type of session will gently introduce patients to the aspects of the sports in a fun and friendly way without enforcing the rules and restrictions of the traditional games. Instructors will be trained in how to deliver the sports as well as important health considerations for the patients of this trial. Those sports included in the trial are offered at the sports facilities during normal timetable hours which will allow patients to continue participating after their 12-week programme.

#### Research questions and hypotheses

The primary purpose of the trial is to test the independent and synergistic efficacy of the 12-week sports-oriented exercise referral intervention and the self-help web-based intervention intended to promote sustained and increased levels of physical activity over a period of 12 months. Do these interventions separately and in combination improve these indicators and do they improve them above that expected by existing gym-based GP referral alone? The secondary questions are whether these interventions improve other clinical indicators of cardiovascular health (for example, body mass index, waist and hip measures, blood pressure and other measures that contribute to the QRisk2 risk indicator) above that expected by existing gym-based GP referral? Do these interventions increase participation in sporting activity over a period of 12 months?

The interventions individually will be regarded as successful if they lead to an average increase in activity of 100 metabolic equivalent of task (MET)-minutes per week. This equates to engaging in an extra moderate intensity activity, say brisk walking (3.3 MET) for 30 minutes per week. The new interventions will be regarded as superior to the standard gym-based referral if they increase activity levels above those of the standard care gym-based exercise by 20 MET-minutes per week.

We will assess changes in indicators relating to cardiovascular health including blood pressure, weight, and waist and hip circumference. In terms of blood pressure, the interventions will be regarded as successful if they lead to an average decrease in blood pressure of 2.5 mmHg. We will assess the proportion of the sample reducing their blood pressure by more than 10 mmHg and an intervention will be regarded as a success if more than 20% of the members of that study arm achieve a reduction greater than this. The interventions will also be deemed to have been successful in promoting sports participation if they increase sports-related activity by 100 MET-minutes per week.

## Methods/design

Participants will be inactive people aged 18 to 74 years who are eligible to be referred by their GPs for an exercise intervention. Participants will be people with hypertension or high-normal blood pressure and assessed as being inactive or moderately inactive using the General Practice Physical Activity Questionnaire (GPPAQ) screening tool. The inclusion and exclusion criteria are detailed below.

The study will be conducted in the Guildford and Waverley CCG area (Surrey, UK) and patients will be recruited via GP surgeries serving catchment areas within reasonable reach of Surrey Sports Park and the five DC leisure centres in Waverley, Surrey, UK. We have designed the study with the intention to allow recruitment for all patients eligible for an exercise referral within the 9 months of the proposed trial data collection phase.

**Figure 1:**
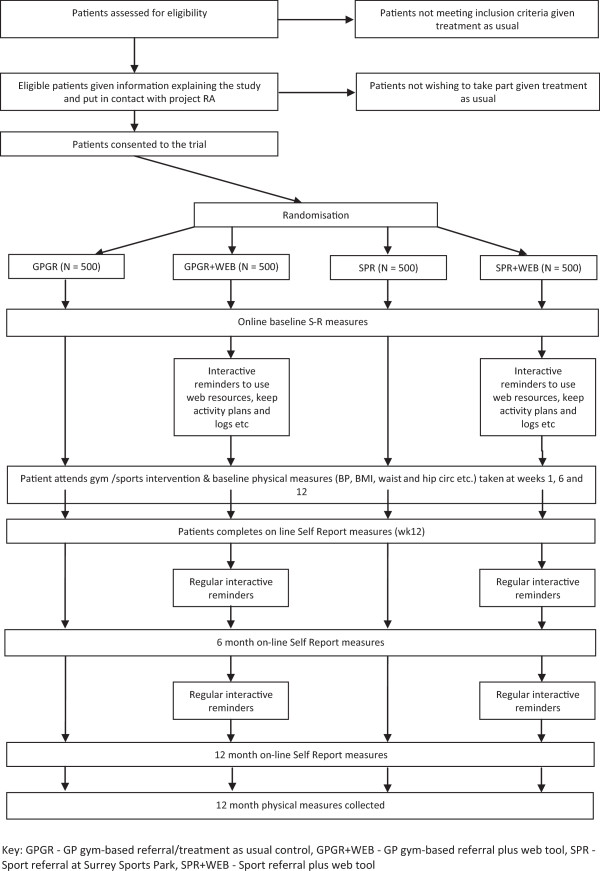
**Trial design diagram.**

The trial design is shown in Figure 1. The key features are that, after a patient has been assessed as likely to benefit from an exercise intervention, their referral is to the project research assistant who will allocate the patient to one of four conditions on the basis of previous independent randomisation. These are: 1) GP gym-based referral (GPGR, the treatment as usual control); 2) GP gym-based referral plus web tool (GPGR + WEB); 3) sport referral at Surrey Sports Park (SPR); and 4) sport referral at Surrey Sports Park plus web tool (SPR + WEB). After obtaining informed consent patients attend a 12-week exercise programme (sport or gym) and their activity levels and sporting participation are monitored again at 6 months and 12 months after starting the exercise programme. Pending ethical approval the intention is to commence recruitment in the early autumn of 2013 for a period of 9 months.

The inclusion criteria are as follows:

1. The patient is aged 18 to 74 years at randomisation.
2. The patient has been diagnosed as having hypertension or high-normal blood pressure as recorded on their GP records and is regarded by their GP as someone who would benefit from attending an exercise programme. High-normal blood pressure is defined as systolic blood pressure 130 to 139 mmHg or diastolic blood pressure 85 to 89 mmHg based on clinical records.
3. The patient is screened as being ‘inactive’ or ‘moderately inactive’ on the GPPAQ.
4. The patient has access to the internet and an email account.
5. The patient is able to understand the Informed Consent Form, and understand study procedures.
6. The patient has signed the Informed Consent Form.

The exclusion criteria are as follows:

1. The patient is pregnant.
2. Inability to freely consent to take part in the study
3. Inability to understand the study materials.
4. The patient is unable to access the internet/email
5. Current participation in another clinical trial relating to physical activity or exercise
6. Any condition that, in the GPs opinion, compromises the subject’s ability to meet protocol requirements or to complete the study.
7. The patient is referred out of the trial by the exercise professional on the grounds that the programme is, in their judgement, likely to cause harm.

### Measures

PA will be assessed via an online questionnaire using the short-form version of the International Physical Activity Questionnaire [29], which is a well validated measure of physical activity in a number of domains and permits the calculation of MET per minute values for activities in each domain, including leisure and sporting activity [30]. As recent research has shown that the International Physical Activity Questionnaire can overestimate activity in sedentary individuals we will use a second validated self-report measure, the Self-Report Walking and Exercise Tables [31] that also permits the estimation of MET minutes. We will include additional items to tap the types of sporting activity people do.

Participants will complete the GPPAQ screening tool at the beginning and end of the study for inclusion on their patient records.

Health indicators will include measures of height and weight (body mass index (BMI)), waist and hip circumference, and blood pressure. These measures will be assessed by the exercise professional at the beginning, middle and end of the 12-week exercise programme. They will be collected again at the end of the 12-month period on visiting their GP surgery. Patients will be asked to consent to the collection of their most recent blood sugar, cholesterol/high-density lipoprotein ratio and indications of whether they are receiving treatment for high blood pressure. We will ask for access to medical records to assess whether they have chronic kidney disease, diabetes, rheumatoid arthritis or atrial fibrillation. These indicators are required for the QRisk2 calculations. To assess safety we will also monitor well-being during the period of the exercise programmes. Blood pressure measurements will be taken by the research team and/or by specially trained exercise professionals using Omron M6W blood pressure monitors (Omron Health Care Co. Ltd, 53, Kunotsubo, Terado-cho, Muko, Kyoto, Japan).

General health will be assessed with the RAND SF36 [32] along with short measures of drinking (the AUDIT [33]) and smoking behaviour (The Fagerstrom Test [34]). These will be assessed at baseline and at 12 month follow-up.

Intervention exposure will be assessed by attendance at the exercise programme sessions. Web-tool usage will be assessed by number and duration of logins over the study period.

Patient evaluation of, and responses to, the interventions will be assessed by a short online questionnaire. This will assess what they felt about the interventions on rating scales, the degree to which assumed mediator variables were influenced by the interventions (for example, perceptions of control and autonomy) and it will collect free-form comments about the interventions. Views about the trustworthiness of web sources of exercise advice will be assessed.

Patient orientation towards activity will be assessed by a short series of questions to assess control beliefs and autonomy over choosing to do physical activity, intentions and motivations.

Demographic and background information will include gender, age, ethnic origin, occupation, marital status and postcode (for assessment of socioeconomic deprivation). They will be asked if there is a family history of premature coronary heart disease. Participants will be asked about their use of GP websites and other online health and well-being sites.

### Sample size and power calculations and randomisation

Davies and colleagues report a meta-analysis of internet-based exercise intervention studies which suggests an average effect size of d = 0.14 [16]. A Cochrane review of general interventions to promote activity among inactive adults reports an average SMD = 0.28, but notes considerable heterogeneity in effects sizes [4]. In the case of internet interventions aimed at reducing blood pressure, effects sizes of the order of SMD = 0.27 and 0.17 for systolic and diastolic blood pressure, respectively (equating to drops of 3.8 and 2.1 mmHg, respectively), have been identified in a recent meta-analysis [35]. Based on these analyses, an effect size of 0.125 can be assumed for comparing means of MET-minutes per week and mmHg between the intervention and control arms (equinumerous) for a two-arm study. With this effect size, if each arm consists of 500 subjects, a power of 80% is obtained for a two-sided statistical contrast to show statistical significance, at the 5% level, of the difference of the means of the two arms with respect to these outcome variables. It is therefore decided that there will be 500 subjects in each of the four arms of the present study.

Block randomisation is to be conducted by the University of Surrey’s Clinical Research Centre. Participants cannot be blinded to the nature of the intervention they are receiving and for reasons of monitoring and contacting participants the research assistant also cannot be blinded. The primary analyst will be blinded to participant allocation.

### Proposed analytic strategy

#### Sample description

The number of participants completing the 12 exercise courses and each of the assessment waves will be tabulated. The number of participants withdrawing during the course of the study will be tabulated by reason for withdrawal where known. The data will be screened to detect outliers and assess the distributions of the primary and secondary outcome variables. Outliers will not be excluded from analyses unless there is independent evidence that their scores are not valid.

Demographic and other patient characteristics (age, sex, weight, height and BMI) recorded on screening will be tabulated by treatment arm. Descriptive statistics will include n, mean, standard deviation, median, minimum and maximum. Differences in baseline levels of outcome variables (activity, BMI, and so forth) will be identified.

#### Intervention effectiveness

For the primary outcome of activity, and assuming normal variable distributions, the intervention arms will be compared to the control arm using *t*-tests and one-way analysis of covariance using study arm as the between-subjects factor and baseline activity scores as the covariate (following Senn, [36]). Similar analyses will be conducted for the secondary outcome variables and in all cases effect sizes reported. Where variable distributions do not initially permit the use of parametric inference tests, variables will be subjected to mathematical transformation to achieve normality. In cases where transformations are not effective, non-parametric tests on difference scores will be used. Differences between arms in the proportions achieving more than 10 mmHg reductions in blood pressure will be assessed using chi-square tests.

Analyses will be conducted on a per-protocol basis. Interim assessment of the efficacy of the interventions will be made at 6 months.

#### Moderator and mediator analyses

To attempt to establish how the interventions work and for whom, we will conduct a series of mediation and moderation analyses following established bootstrapping procedures outlined in [37]. Putative moderators will include participant demographic factors, baseline intentions to engage in activity, feelings of self-efficacy and autonomy.

Potential mediators will include number of implementation intentions formed (in web-tool arms), frequency and intensity of use of the web-based tool, enjoyment of the 12-week exercise programme, attendance at the programme, perceptions of autonomy over exercise during the training programme, and intentions to exercise post-exercise programme.

### Ethics

The trial has received a favourable ethical opinion by the London Bloomsbury National Research Ethics Service committee (13/LO/1170) and commenced recruitment in October 2013. To date, ten practices have expressed an interest in identifying potential participants for the trial.

## Discussion

This randomised controlled trial compares GP referral to sport-based exercise with referral to the more conventional gym-based exercise programme. It hypothesises that enjoyment, choice and therefore some degree of control might result in greater persistence. It is needed because, currently, such a small proportion of people referred for exercise persist. The challenges for this trial include recruiting sufficient numbers to the trial, and matching those who are recruited to appropriate exercise-based interventions. We need to recruit 2,000 patients and we need to provide those allocated to sports-based exercise activities at a time and of a type that they might opt to participate in. There are many uncertainties as to the feasibility of this, which will be tested in the course of this study.

Further parallel work is required to explore change in vascular risk and the potential cost effectiveness of sport-based exercise interventions. The trial is not looking at overall cardiovascular risk, or change in risk score (such as Framingham or QRisk score), nor at major cardiovascular outcomes and death. The former may be required in order to carry out a cost-effectiveness analysis. The latter would provide data on the safety of referral for sport-based exercise programmes and also about any change in health outcomes, though the scale of the trial and duration of follow-up would not provide sufficient data for any difference to be detectable between arms.

The theoretical framework around the importance of enjoyment and control potentially leading to greater persistence with sport-based, compared to gym-based, exercise may be challenged if we find no difference between arms.

## Trial status

The trial commenced in October 2013 and data collection is continuing.

## Abbreviations

BMI: body mass index
GP: general practitioner
GPGR: GP gym-based referral
GPGR + WEB: GP gym-based referral plus web tool
GPPAQ: General Practice Physical Activity Questionnaire
MET: metabolic equivalent of task
PA: physical activity
SPR: sport referral at Surrey Sports Park
SPR + WEB: sport referral at Surrey Sports Park plus web tool.
